# Alcohol and drug consumption among motor vehicle drivers in the Brittany region of France: A 9-year cross-sectional population study

**DOI:** 10.1016/j.pmedr.2021.101454

**Published:** 2021-06-17

**Authors:** Brendan Le Daré, Adeline Degremont, Clémence Couty, Alain Baert, Renaud Bouvet, Isabelle Morel, Thomas Gicquel

**Affiliations:** a Rennes University Hospital, Forensic Toxicology Laboratory, F-35000 Rennes, France; b Univ. Rennes, INSERM, INRA, CHU Rennes, Institut NuMeCan (Nutrition, Metabolism and Cancer), F-35000 Rennes, France; c Rennes University Hospital, Pharmacovigilance, Pharmacoepidemiology and Drug Information Centre, Department of Clinical Pharmacology, F-35033 Rennes, France; d Univ Rennes, EA 7449 REPERES ‘Pharmacoepidemiology and Health Services Research’, F-35000 Rennes, France; e Rennes University Hospital, Department of Forensic Medicine, F-35000 Rennes, France; f Univ. Rennes, EA IDPSP – UR1_RS438, F-35000 Rennes, France

**Keywords:** Prevention, Policy, Public health, Motor vehicle, Cross sectional study, Descriptive epidemiology

## Abstract

The primary objective of the present study was to evaluate the frequency of positive tests for alcohol and drugs during roadside testing or after road accidents among drivers in the Brittany region of France. The study’s secondary objective was to describe the blood concentrations of the substances found during these tests, in order to provide a scientific basis for the establishment or modification of legislative threshold values for road injuries prevention. We performed a cross-sectional study of a database compiled by Rennes University Hospital’s toxicology laboratory in the Brittany region of France between 2010 and 2018. Driver’s age, sex, and test status (positive or negative), and blood levels of ethanol, 9-tetrahydrocannabinol (THC), methylene dioxymethamphetamine (MDMA), amphetamine, benzoylecgonine and 6-monoacetylmorphine (6-MAM) were collected. Twelve thousand four hundred and ninety-seven drivers (males: 86.1%; median (range) age: 29 (15–94)) have provided roadside blood samples, giving a total of 25,998 test results. Among the 10,996 drivers with at least one positive test, the median blood concentrations of ethanol, THC, MDMA, amphetamine, benzoylecgonine, and 6-MAM were respectively 1.82 g/L, 2.41 ng/mL, 138.4 ng/mL, 67.7 ng/mL, 173.3 ng/mL, and 0.97 ng/mL. 1159 (10.54%) of the 10,996 drivers tested positive for two or more substances, and 151 (1.4%) tested positive for three or more substances. With the exception of heroin, the currently recommended threshold values appear to be appropriate for road injuries prevention with regard to the concentrations observed in offenders.

## 1 Introduction

According to a World Health Organization (WHO) report published in 2018, 27% of road traffic deaths worldwide are attributable to alcohol; this represented 373,000 deaths in 2016, including 187,000 people other than the driver (World Health Organization, 2018). The risk of accident increases rapidly and exponentially with the blood alcohol concentration - even at low doses (Taylor and Rehm, 2012). In France, driving under the influence of alcohol (DUIA) is the second leading cause (after speeding) of fatal road crashes; it accounted for 18% of the 3503 deaths in 2018 (Observatoire national Interministériel de la sécurité routière, 2018). Although drink-driving campaigns are frequent and prominent, the general public is less aware of the impact of driving under the influence of drugs (DUID) on fatal road crashes. The consequences of DUID are more difficult to identify than those of DUIA - particularly because of the difficulty in (i) confirming blood screening results for the drug substance using high-performance equipment (i.e. chromatographic separation coupled to mass spectrometry) and (ii) establishing a causal relationship between a drug concentration threshold and an impairment in driving skills. Furthermore, the risk of accident varies according to the type of drug and whether or not other psychoactive substances are used concomitantly (World Health Organization, 2018). Data from the USA show that in 2016; 43.6% of the drivers responsible for fatal road injuries were positive for at least one narcotic substance (Governors Highway Safety Association, 2018).

One of the objectives of criminal sanctions is to discourage DUIA/DUID. Like almost a hundred countries around the world, France has introduced a per se legislation that sets a maximum tolerated blood alcohol concentration for driving (0.5 g/L, reduced to 0.2 g/L for novice drivers and public transport drivers) (Code de la route, 2019). In the field of narcotics, the French Highway Code punishes driving under the influence of narcotics as soon as a test proves this (i.e. zero tolerance, according to *per se* legislation) (Bouvet et al., 2015). At present, the law is limited to four families of illicit substances: cannabinics (9-tetrahydrocannabinol (THC)), amphetaminics (such as amphetamine, methylene dioxymethamphetamine (MDMA)), 3,4-methylenedioxyamphetamine (MDA), 3,4-methylenedioxy-N-ethylamphetamine (MDEA) and methamphetamine), cocainics (such as cocaine and its metabolite benzoylecgonine), and opiates (such as morphine and 6-mono acetylmorphine (MAM)) (Arrêté du 13 décembre, 2016). According to French legislation, the threshold blood concentration for a positive test is 0.5 ng/mL for cannabinics and 10 ng/mL for the other three families (Arrêté du 13 décembre, 2016).

Assays for these psychoactive substances are requested by the traffic police during random roadside testing or after a crash has occurred. The primary objective of the present study was evaluate the frequency of positive tests for alcohol and drugs during roadside testing or after road accidents among drivers in the Brittany region of France between 2010 and 2018. The secondary objective was to describe the blood concentrations of substances found during these tests, in order to provide a scientific basis for the establishment or modification of legislative thresholds for road injuries prevention.

## 2 Methods

### 2.1 Data source and design

Following a request by the police, Rennes University Hospital’s toxicology laboratory carries out assays on blood samples taken (i) after a crash, (ii) in the context of checkpoints (such as raves and festivals) or (iii) during random roadside testing, at any time of day and any location. In the last two cases, blood samples were taken after positive oral fluid test or alcohol tester. Since the proportion of each of these three origins is unknown, we pooled the results together. The requested drug screens include ethanol, the cannabinoid THC, MDMA, other amphetamines, the cocaine metabolite benzoylecgonine, and the heroin metabolite 6-MAM. For each sample, several data are recorded in the toxicology laboratory’s database: the tested person’s sex and date of birth, the date and place of the roadside testing or the crash, the substances sought (ethanol, THC, MDMA, amphetamine, benzoylecgonine, and/or 6-MAM), the date of the analytical result, the result (positive or negative), and the blood concentration of analyte in positive tests. Although morphine has been assayed in the laboratory, there is still uncertainty as to the source of this substance; morphine can come either from medical care (morphine or codeine) or from narcotics. In order to be sure of the narcotic intake, we chose to keep only 6-MAM in this study.

We conducted a cross-sectional study of data extracted from the laboratory’s database for the period between January 1st, 2010, and November 22nd, 2018.

### 2.2 Drug testing assessments

The psychoactive substances were obligatorily assayed using the analytical methods set in the French Highway Code (Arrêté du 13 décembre, 2016). Alcohol was quantified using headspace gas chromatography with flame ionization detection (TRACE 2000, Thermo Scientific, San Jose, CA, USA) and an autosampler (TriPlus Headspace, Thermo Scientific). Drugs were assayed using liquid chromatography coupled with mass spectrometer (Q-Exactive™ Orbitrap (since 2014) or TSQ Quantum Ultra, Thermo Scientific) coupled to a pump (Accela 1250, Thermo Scientific), as described elsewhere (Gicquel et al., 2016, Gicquel et al., 2013, Le Daré et al., 2019). The limits of quantification (LOQ) for the different analyses are shown in Table 3.

### 2.3 Statistical analysis

**Fig. 1:**
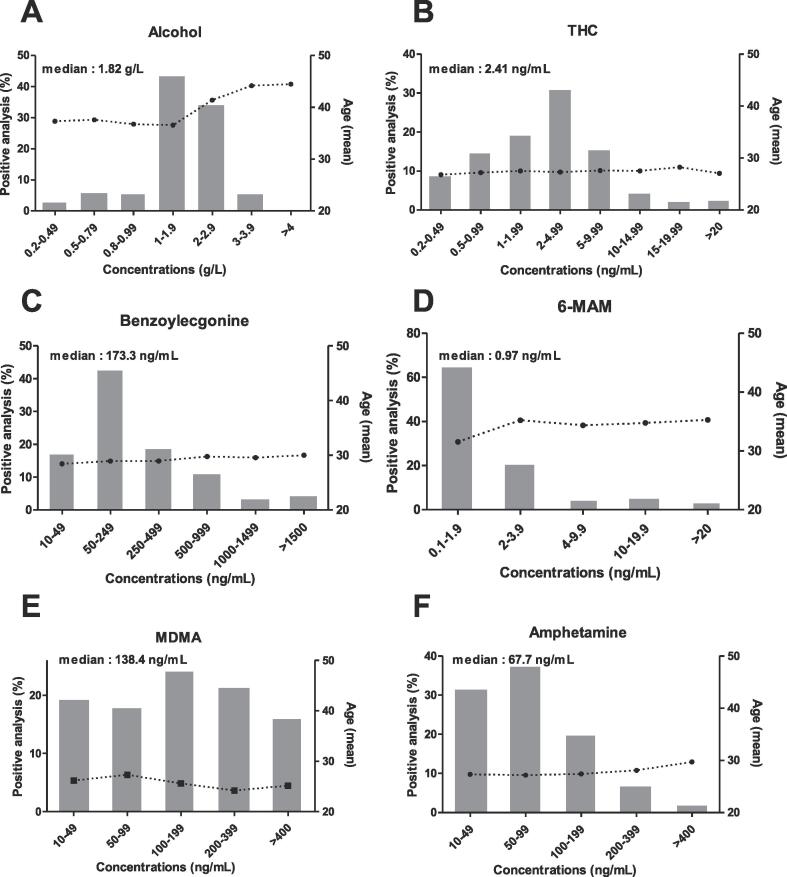
Distributions of the concentrations of psychoactive substances detected (histograms), together with the driver’s mean age (black circles): (A) alcohol (B) THC (C) benzoylecgonine (D) 6-monoacetylmorphine (6-MAM) (E) methylene dioxymethamphetamine (MDMA) (F) Amphetamine.

We analyzed (i) the dataset as a whole, (ii) each substance class, and (iii) samples that were positive for two or more substances. All analyses were performed using SAS software (version 9.4, SAS-institute, Cary, NC, USA) and SAS Enterprise Guide®. Quantitative variables (age and plasma concentration) were described as the mean, median, standard deviation (SD), range and interquartile range (IQR), and qualitative variables (sex, assay request, assay positivity, multiple substance abuse) were described as the number (percentage). Fig. 1 was created using Prism software (version 5.0, GraphPad Software, La Jolla, CA, USA).

## 3 Results

Between 2010 and 2018, a total of 25,998 toxicological analysis results (concerning 12,497 individuals) were logged in the laboratory’s database. The general characteristics of the tested drivers and the requests for alcohol and drug tests are summarized in Table 1. The age of the tested individuals ranged from 15 to 94, with a median of 29 and an IQR of 23 to 38. Over this 9-year period, most of the tested subjects fell into the 15–24 and 25–34 age groups (corresponding respectively to 37.8% and 31.1% of the 25,998 requests for analyses). The male/female sex ratio was 6.5 (NB: the sex was not reported in 0.2% of cases). Cannabis and alcohol were the most frequently tested-for substances during roadside testing; they accounted for 33% and 20.3% of the assay requests, respectively (Table 1).

**Table 1:** Characteristics of the 12,497 motor vehicle drivers and 25,998 drug test requests recorded between 2010 and 2018. The data on each substance are quoted as the number (%).

Driver characteristics	Alcohol	Cannabis	Amphetaminics	Cocaine	Heroin	Total
Age (years)						
15–24	1297 *(24.5%)*	3770 *(43.9%)*	1807 *(41.1%)*	1629 *(38.4%)*	1319 *(37.7%)*	9822 *(37.8%)*
25–34	1263 *(23.9%)*	3021 *(35.2%)*	1346 *(30.6%)*	1381 *(32.5%)*	1069 *(30.6%)*	8080 *(31.1%)*
35–44	1014 *(19.2%)*	1104 *(12.9%)*	638 *(14.5%)*	636 *(15.0%)*	564 *(16.1%)*	3956 *(15.2%)*
45–54	848 *(16.0%)*	391 *(4.6%)*	323 *(7.4%)*	319 *(7.5%)*	295 *(8.4%)*	2176 *(8.4%)*
55–64	484 *(9.2%)*	153 *(1.8%)*	145 *(3.3%)*	143 *(3.4%)*	131 *(3.7%)*	1056 *(4.1%)*
> 65	381 *(7.2%)*	136 *(1.6%)*	134 *(3.1%)*	134 *(3.2%)*	123 *(3.5%)*	908 *(3.5%)*

Sex						
Male	4341 *(82.1%)*	7670 *(89.4%)*	3760 *(85.6%)*	3622 *(85.4%)*	2991 *(85.5%)*	22,384 *(86.1%)*
Female	939 *(17.8%)*	880 *(10.3%)*	622 *(14.2%)*	613 *(14.4%)*	502 *(14.3%)*	3556 *(13.7%)*
Missing	7 *(0.1%)*	25 *(0.3%)*	11 *(0.2%)*	7 *(0.2%)*	8 *(0.2%)*	58 *(0.2%)*
Total drivers per substance	**5287**	**8575**	**4393**	**4242**	**3501**	**25 998***(100%)*
Percentage of test requests	*20.3%*	*33%*	*16.9%*	*16.3%*	*13.5%*	

We described the general characteristics of the drivers with positive tests (Table 2), performed a statistical analysis of the assay results (Table 3), and analyzed the distributions of concentration ranges as a function of the driver’s age (Fig. 1).

**Table 2:** Characteristics of the population testing positive for one or more substances during roadside sampling. The percentages in brackets refer to the proportion for each substance.

	Alcohol	Cannabis	Amphetaminics	Cocaine	Heroin	Total
Positive tests						
N	**4347**	**5361**	**571**	**634**	**83**	**10,996**
*Proportion of drivers with a positive test*	*82.2%*	*62.5%*	*13%*	*14.9%*	*2.4%*	*42.3%*

Age (years)						
15–24	1008 *(23.2%)*	2526 *(47.1%)*	322 *(56.4%)*	211 *(33.3%)*	8 *(9.6%)*	4075 *(15.7%)*
25–34	1069 *(24.6%)*	2133 *(39.8%)*	199 *(34.9%)*	325 *(51.3%)*	48 *(57.8%)*	3774 *(14.5%)*
35–44	887 *(20.4%)*	595 *(11.1%)*	43 *(7.5%)*	84 *(13.2%)*	23 *(27.8%)*	1632 *(6.3%)*
45–54	739 *(17%)*	101 *(1.9%)*	7 *(1.2%)*	14 *(2.2%)*	4 *(4.8%)*	865 *(3.3%)*
55–64	399 *(9.2%)*	5 *(0.1%)*	0	0	0	404 *(1.6%)*
>65	245 *(5.6%)*	1 *(0.02%)*	0	0	0	246 *(0.9%)*

Sex						
Male	3632 *(83.6%)*	4957 *(92.5%)*	482 *(84.4%)*	545 *(86.0%)*	74 *(89.2%)*	9690 *(88.1%)*
*Positivity rate among males*	*83.7%*	*64.6%*	*12.8%*	*15%*	*2.5%*	*43.3%*
Female	711 *(16.4%)*	385 *(7.2%)*	86 *(15.1%)*	89 *(14.0%)*	8 *(9.6%)*	1279 *(11.7%)*
*Positivity rate among female*	*75.7%*	*43.7%*	*13.8%*	*14.5%*	*1.6%*	*36%*
Missing data for sex	4 *(0.1%)*	19 *(0.3%)*	3 *(0.5%)*	0	1 *(1.2%)*	27 *(0.2%)*

**Table 3:** Positive tests for psychoactive substances recorded between 2010 and 2018. LOQ: limit of quantification in our laboratory; IQR: interquartile range; THC: 9-tetrahydrocannabinol BZE: benzoylecgonine; MDMA: methylene dioxymethamphetamine; 6-MAM: 6-mono acetylmorphine.

	LOQ	Median	IQR	range	Mean ± SD	N
Alcohol (g/L)	0.2	1.82	1.30–2.37	0.20–5.24	1.84 *(±0.76)*	**4347**
THC (ng/mL)	0.20	2.41	1.05–5.01	0.20–109.50	4.32 *(±6.61)*	**5361**
BZE (ng/mL)	10	173.3	72.0–417	10–5991	380.6 *(±617.9)*	**634**
MDMA (ng/mL)	10	138.4	65.5–295	10–1920	227.6 *(±254.8)*	**436**
Amphetamine (ng/mL)	10	67.7	39.1–119.2	10–1239	103.8 *(±137.3)*	**195**
6-MAM (ng/mL)	0.10	0.97	0.37–2.9	0.11–31.80	2.88 *(±5.29)*	**83**

As in the study population as a whole, the great majority of the positive assays (83.6%) came from male drivers; this was true for all substances (Table 2). However, the positivity rates for the two sexes were similar: 43.3% of the men and 36% of the women. The age profile varied with the substance detected. Most of the amphetaminics and cannabis users were aged between 15 and 24 (56.4% and 47.1%, respectively), while most of the cocaine and heroin users were aged between 25 and 34 (51.3% and 57.8% respectively). Over the age of 55, almost all of the positive results concerned alcohol consumption (Table 2). Furthermore, the highest blood alcohol concentrations (BACs) (>2 g/L) were mainly observed in older drivers (over the age of 40); this age profile was not found with other substances (Fig. 1). In general, the requests for these substances (alcohol + narcotics) mainly concerned young people: 68.9% of the tests were carried out on drivers under the age of 35. With regard to alcohol, 32.4% of the requests concerned people over 45 years of age (Table 1). Taken as a whole, these results emphasize that the test requests matched the user profiles.

The highest positive test rates were observed for alcohol and cannabis (82.2% and 62.5%, respectively) (Table 2). With regard to alcohol, almost half the individuals (43.8%) had a BAC between 1 and 2 g/L (median: 1.82 g/L) (Fig. 1; Table 3). The median concentration of THC (attesting to driving under the influence of cannabis) was 2.41 ng/mL, and 50.7% of the values fell in the range from 1 to 5 ng/mL (Fig. 1, Table 3). The positive test rate for cocaine was 14.9%. Although most of the blood benzoylecgonine concentrations fell in the range from 50 to 250 ng/mL, the median value was 173.3 ng/mL with a range from 10 to 5991 ng/mL - indicating a broad dispersion (Table 3, Fig. 1). Similarly, the MDMA and amphetamine varied greatly from one driver to another, with median values of 138.4 ng/mL (range from 10 to 1920 ng/mL) and 67.7 ng/mL (range from 10 to 1239 ng/mL), respectively (Table 3). Lastly, opiates were the least frequently sought-after drug (13.5% of the total), and the 6-MAM assay had the lowest positivity rate (2.4%) (Table 1, Table 2). The blood 6-MAM concentrations were also very low, with a median value of 0.97 ng/mL. Only 8.6% of positive 6-MAM assays gave a value above 10 ng/mL (Table 3, Fig. 1). Overall, our results show that the drivers’ blood concentrations were well above our laboratory's corresponding LOQs (Table 3).

The abuse of multiple psychoactive substances (Table 4) appeared to be frequent, although the pattern varied according to the type of product consumed. Overall, 10,996 of the 12,487 drivers (87.98%) were positive for at least one of the prohibited substances (Table 4). Multiple substance abuse was observed among 12.9%, 23.4%, 70.4%, 95.3%, and 69.9% of the drivers testing positive for alcohol, THC, amphetaminics (MDMA and/or amphetamine), cocaine and heroin, respectively. Of the 10,996 individuals who were positive for at least one substance, 1159 (10.54%) were positive for two or more substances, and 151 (1.37%) were positive for three or more substances. Only six individuals were positive for four substances in the same blood sample (Table 4). Details of the combinations of substances detected in individuals with two or three positive tests for the same blood sample are presented in the Supplementary Data.

**Table 4:** Multiple substance abuse among our population of drivers between 2010 and 2018. ^a^ Details of the combinations of substances detected in individuals with 2 or 3 positive tests for the same blood sample are presented in the Supplementary Data.

Drug category	N	% of the total population (n = 12497)	% of the positive population (n = 10996)	% of multiple substance abusers (n = 1159)
**None**	**2817**	***22.54%***	***–***	*–*
**A single drug**	**8521**	***68.18%***	***77.49%***	*–*
Alcohol	3850	*30.80%*	*35.01%*	*–*
Cannabis	4292	*34.34%*	*39.03%*	*–*
Amphetamines	167	*1.34%*	*1.52%*	*–*
Cocaine	178	*1.42%*	*1.61%*	*–*
Heroin	34	*0.27%*	*0.31%*	*–*
**Two drugs*^a^***	**1008**	***8.06%***	***9.17%***	***86.97%***
Cannabis + alcohol	399	*3.19%*	*3.63%*	*34.43%*
Cannabis + amphetamines	261	*2.08%*	*2.37%*	*22.52%*
Cannabis + cocaine	242	*1.94%*	*2.20%*	*20.88%*
Others combinations	106	*0.85%*	*0.97%*	*9.14%*
**Three drugs*^a^***	**145**	***1.16%***	***1.32%***	***12.51%***
Cannabis + cocaine + alcohol	42	*0.34%*	*0.38%*	*3.62%*
Cannabis + cocaine + amphetamines	76	*0.61%*	*0.69%*	*6.56%*
Cannabis + cocaine + heroin	12	*0.10%*	*0.11%*	*1.04%*
Other combinations	15	*0.10%*	*0.14%*	*1.29%*
**Four drugs**	**6**	***0.04%***	***0.05%***	***0.52%***
**One or more drugs**	**10,996**	***87.98%***	***100%***	*–*
**Two or more drugs**	**1159**	***9.27%***	***10.54%***	***100%***
**Three or more drugs**	**151**	***1.21%***	***1.37%***	***13.03%***

## 4 Discussion

The results of this large, cross-sectional analysis of data collected between 2010 and 2018 by Rennes University Hospital’s toxicology laboratory provides an interesting overview of DUIA and DUID in the Brittany region of France. >40% of the blood assays were positive for at least one psychoactive substance - mainly ethanol and cannabis. To the best of our knowledge, the present study is the first of this magnitude to have explored the patterns of psychoactive substance consumption (including multiple substance abuse) among drivers in France, the consumption profiles, and the blood concentrations.

The great majority of the blood tests assessed in the present study (86.5%) concerned men. In the literature, male sex is associated with more time spent driving, a greater frequency of speeding violations, and greater alcohol consumption (relative to females) (Norris et al., 2000). Along with this overall male predominance, it is noteworthy that the positivity rates for a given substance were similar for men and women. We did not observe an association between age and the blood concentration of narcotics (cannabis, cocaine, opiates and amphetaminics). Blood alcohol concentrations above 2 g/L were mainly observed in drivers over the age of 40. In the literature, similar results have been observed in Sweden, where the highest roadside BACs were found in the 40 to 55 age class among both men and women (Jones and Holmgren, 2009). These results might be explained by (i) changes in ethanol metabolism with advancing age because activity of the enzymes involved, such as alcohol and acetaldehyde dehydrogenase and cytochrome P450 2E1, diminish with age (Meier, 2008) and (ii) an increase in alcohol consumption among older adults observed in recent decades (Calvo et al., 2020).

According to a report from the European Monitoring Centre for Drugs and Drug Addiction (EMCDDA), alcohol is the most commonly used psychoactive substance among drivers in Europe, and cannabis is the most commonly used narcotic (Verstraete and Legrand, 2014). Our results were in line with these data, with a high positivity rate for alcohol (82.2%). In France, the maximum authorized BAC is 0.5 g/L. A BAC above 0.8 g/L leads to more severe sanctions (a court summons) (Code de la route, 2019a, Code de la route, 2019b). In the present study, the BACs of most of the 4,347 alcohol-positive drivers far exceeded the legal threshold, with an average of 1.84 g/L. Hence, 90.7% of the alcohol-positive subjects were liable to severe sanctions (>0.8 g/L blood), and only 6.1% had a BAC between 0.5 g/L and 0.8 g/L. In Sweden, an observational study of 32,814 drivers gave similar results, with an average BAC of 1.74 g/L (Jones and Holmgren, 2009). The results of the DRUID project on DUID, alcohol and medications in four European countries found that 15.1% to 38.9% of the drivers involved in fatal road crashes tested positive for alcohol alone (≥0.1 g/L); the median BAC ranged from 1.1 g/L to 1.8 g/L (depending on the country), and 87% of the drivers had a BAC over 0.5 g/L (Legrand et al., 2014). In our study, the high BACs undoubtedly corresponded to an impaired ability to control the vehicle.

The most frequently detected narcotic in our study was THC, with a positivity rate of 62.5%. The consumers were predominant young males, as previously reported in France (Beck et al., 2017). The highest proportion of male adolescents having experimented with cannabis was observed in the Brittany region. (Splika et al., 2017, Beck, 2017). We measured a mean blood THC concentration of 4.32 ng/mL. In a Swedish study of drivers who had used cannabis, the mean concentration was 2.1 ng/mL (Jones et al., 2008). From an analytical point of view, Jones et al. pointed out that blood THC levels when driving would be much higher than those at the time when the blood sample was collected (Jones et al., 2008). Furthermore, the plasma concentration of inhaled THC drops significantly before the drug’s maximal psychotropic effects are observed (Chiang and Barnett, 1984, Ohlsson et al., 1980). Although epidemiological studies have observed an elevated risk of crashes for whole blood THC concentrations above 1 or 2 ng/mL (Kuypers et al., 2012, Laumon et al., 2005, Gadegbeku et al., 2011), it is therefore not easy to rigorously establish a threshold concentration for impaired driving performance at the time of sampling and include it in legislation. In France, the value of 0.5 ng/mL defined in the current legislation is only a threshold for analytical performance, and not the value above which the driver is convicted (Legifrance, 2016). An expert laboratory’s analytical performance will exceed these requirements and thus it is possible to detect, measure and report a THC concentration below this value (Bouvet et al., 2012). Our analytical performance has enabled us to detect THC at the LOQ of 0.2 ng/mL. Thus, nearly 10% of the drivers had blood concentrations between 0.2 and 0.5 ng/mL and would not have been considered to be positive by the current legislation (Bouvet et al., 2015, Bouvet et al., 2012, Bouvet et al., 2013).

According to the literature data, the prevalence of heroin experimentation among 18–64 year olds in France is low, with an estimated value of 1.5% in 2017 (Drug workbook, 2017). It is therefore not surprising that only 86 of the drivers in our study were positive for 6-MAM. Our laboratory’s analytical performance gave an LOQ of 0.1 ng/mL for 6-MAM; this appears to be adequate, given that the positivity threshold in France is 10 ng/mL (Legifrance, 2016). Our results showed that 91.2% of the heroin consumers had a blood 6-MAM concentration of between 0.1 and 10 ng/mL. Therefore, the majority of heroin-using drivers would not have been convicted, according to the current legislation.

With regard to cocaine, the literature data show a fourfold increase in consumption in France over two decades (from 1.2% in 1995 to 2.6% in 2005, 3.8% in 2010 and 5.6% in 2014) (Beck et al., 2014), which corroborates the EMCDDA data (Monitoring the supply of cocaine to europe - European Monitoring Centre for Drugs and Drug Addiction, 2019). Furthermore, Brittany is one of the regions with the highest consumption of this narcotic in France (Observatoire Français des drogues et toxicomanies, 2019). Around 15% of the drivers in our study tested positive for benzoylecgonine, with blood concentrations that varied markedly (mainly between 50 and 500 ng/mL). Our results corroborate Rooney et al.’s (2002) report on cocaine use among drivers in the UK, in which 51.4% of the individuals had a blood benzoylecgonine concentration of between 100 and 500 ng/mL (Rooney et al., 2016). Hence, the benzoylecgonine concentrations measured in practice are well above the legal threshold of 10 ng/mL, which therefore appears to be appropriate with regard to our current state of knowledge.

Regarding amphetamines, none of the drivers in our study tested positive for methamphetamine, MDEA or MDA. Although epidemiological studies tend to show an increase in methamphetamine seizures in Europe over recent years, these substances are infrequently used in France; this might explain our results (European Monitoring Centre for Drugs and Drug Addiction, 2009). In line with the literature, we found that MDMA use (n = 436) was more frequent than amphetamine use (n = 195). According to Beck et al. (Beck et al., 2014), 4.3% and 2.3% of French people aged 16 to 64 have already experimented with MDMA and amphetamine, respectively (Beck et al., 2017). Although the literature findings are somewhat contradictory, there does not appear to be a dose–effect relationship for impaired driving performance following amphetaminic consumption. (Gustavsen et al., 2006, Lia et al., 2009, Musshoff and Madea, 2012). However, driving under the influence of amphetamines is associated with an elevated risk of accident (odds ratio [95% confidence interval: 12.8 [3.0–54.0]) (Dussault et al., 2002). *Per se* legislation therefore appears to be relevant. In view of our results for the median blood concentrations of MDMA and amphetamine (138.4 ng/mL and 67.7 ng/mL, respectively), the minimum regulatory threshold of 10 ng/mL appears to be appropriate.

In the context of impairment-type legislation, one can question the relevance of regulatory thresholds for narcotic drugs. The literature data on impaired driving skills under the influence of narcotics vary considerably from one country to another (Kuypers et al., 2012, Vindenes et al., 2012, Vindenes et al., 2014, Hargut et al., 2011). Our results highlighted frequent multiple substance abuse in the Brittany region; this is likely to accentuate an impairment of driving skills and thus increase the risk of road injuries (Dussault et al., 2002). A repressive approach based on threshold concentrations does not generally take multiple substance abuse into account. Although Canada has built an unusual legislative framework for multiple substance abuse (Government of Canada D of J. Impaired Driving Laws, 2018), this approach is primarily limited by (i) strong inter-individual variations in impaired driving performance, and (ii) the absence of data on the multiple combination patterns found in practice (Supplementary Data). The French legislation is strict, in view of the “zero tolerance” approach that it applies to narcotic drugs. However in this study, we found drug concentrations well above the thresholds considered as causing an alteration in driving ability. In this context, our present results suggest that there would be no conviction difference between *per se* legislation on one hand and impairment-type legislation based on a threshold set (apart from 6-MAM) on the other. “Zero tolerance” is a clear message to drivers and is coherent from a legal standpoint: the law cannot establish an offence and then leave it unpunished.

### 4.1 Limitations

Our findings must be interpreted in the context of three potential limitations.

Firstly, the number of BAC requests was probably underestimated, since a blood alcohol test is only requested if it is impossible to obtain a valid test result in expired air or if the driver cannot or will not blow into the test device. Given that these tests are targeted, ethanol was not the most frequently requested analyte. However, it was associated with a high positivity rate (82.2%).

Secondly, we decided to analyse tests for 6-MAM (a specific heroine metabolite) and not tests for morphine (a non-specific heroine metabolite) because of the difficulty of discriminating between opioid misuse on one hand and the medical use of morphine on the other (Maas et al., 2018). Hence, we probably therefore slightly underestimated the positivity rate for opiates, as cases of low 6-MAM and morphine > 10 ng/mL can occur. Also, we have chosen not to include cocaine tests in view of the very low number of positive cases found. Since this substance is very rapidly metabolized to benzoylecgonine, we think it is more relevant to keep only the positive tests for this metabolite. This includes the risk of missing cases with high cocaine and low benzoylecgonine.

Thirdly, our research was dependent on test requests by law enforcement officers; multiple substance abuse may therefore have been underestimated. Furthermore, a study of French and Belgian drivers showed that many new psychoactive substances are found in combination with other commonly abused drugs. In 2018, Wille et al showed that the positivity rate for new psychoactive substances was 7% in blood samples obtained during roadside testing in Belgium, and was 11% in oral fluid samples obtained from negatively screened test pads (Wille et al., 2018). Lastly, other high-risk products (such as fentanyl, benzodiazepines and related products) are also frequently consumed by drivers. Although this consumption increases the risk of road injuries, these substances are not prohibited by the French Highway Code; hence, the corresponding tests are very rarely requested by the police (Kriikku et al., 2015, Pergolizzi et al., 2018, Valen et al., 2019).

## 5 Conclusion

Overall, 10,996 of the 12,487 drivers (87.98%) were positive for at least one of the prohibited substances. Levels of multiple substance abuse in Brittany are high. Our results also show that the thresholds set out in the current legislation are appropriate (with the exception of that for heroin). As the threshold concentrations set under impairment-type legislation (i) vary widely from one country to another, and (ii) rarely take the issue of multiple substance abuse into account, an impairment-based approach does not necessarily appear to be relevant to road injury prevention. Repression based on proof of the consumption of one or more illegal substances while the driver is using his/her vehicle has the advantage of being clear and readily understandable. However, France’s strict legislation limits academic research on the substances discussed here – thus slowing identification of the large number of other psychoactive drugs likely to functionally impair a person’s driving ability.

## Funding

This research did not receive any specific grant from funding agencies in the public, commercial or not-for-profit sectors.

## CRediT authorship contribution statement

All authors contributed significantly, and all authors agree with the content of the manuscript. **Brendan Le Daré:** Methodology, Collection and/or assembly of data, Data analysis and interpretation, Manuscript writing, Final approval of manuscript. **Adeline Degremont:** Methodology, Collection and/or assembly of data, Data analysis and interpretation, Manuscript writing, Final approval of manuscript. **Clémence Couty:** Conceptualization, Data analysis and interpretation, Final approval of manuscript. **Alain Baert:** Final approval of manuscript. **Renaud Bouvet:** Final approval of manuscript. **Isabelle Morel:** Conceptualization, Final approval of manuscript. **Thomas Gicquel:** Conceptualization, Methodology, Collection and/or assembly of data, Data analysis and interpretation, Manuscript writing, Final approval of manuscript.

## Declaration of Competing Interest

The authors declare that they have no known competing financial interests or personal relationships that could have appeared to influence the work reported in this paper.
