# Supplementary material for: Alcohol and drug consumption among motor vehicle drivers in the Brittany region of France: A 9-year cross-sectional population study
Source: Prev Med Rep. 2021 Jun 17;23:101454. doi: 10.1016/j.pmedr.2021.101454 (PMC8227838; doi:10.1016/j.pmedr.2021.101454)
Supplement: Supplementary Data 1 [file mmc1.docx]

**Supplementary Data. Table 5: combinations of substances detected in individuals with two or three positive tests for the same blood sample. For each substance, data are expressed as the number and then as a percentage of the number of drivers who consumed two or three substances. By way of an example: “N = 399 (80.93%)” for alcohol + cannabis consumption means that the 399 drivers with alcohol + cannabis accounted for 80.93% of those who had consumed alcohol plus one or two other substances.**

| **Drug** | **2^nd^ combined drug** | **N *(%)*** | **3^rd^ combined drug** | **N *(%)*** |
| --- | --- | --- | --- | --- |
| **Alcohol** | None | 3850 | - | |
|  | Cannabis | 399 *(80.93%)* | Cocaine | 42 *(8.52%)* |
|  |  |  | Amphetamines | 10 *(2.03%)* |
|  |  |  | 6-MAM | 1 *(0.20%)* |
|  | Cocaine | 29 *(5.88%)* | Cannabis | 42 *(8.52%)* |
|  |  |  | Amphetamine | 3 *(0.61%)* |
|  | Amphetamine | 6 *(1.22%)* | Cannabis | 10 *(2.03%)* |
|  |  |  | Cocaine | 3 *(0.61%)* |
|  | 6-MAM | 3 *(0.61%)* | Cannabis | 1 *(0.20%)* |
| **Cannabis** | None | 4292 | - | |
|  | Alcohol | 399 *(37.53%)* | Cocaine | 42 *(3.95%)* |
|  |  |  | Amphetamine | 10 *(0.94%)* |
|  |  |  | 6-MAM | 1 *(0.09%)* |
|  | Amphetamine | 261 *(24.55%)* | Cocaine | 76 *(7.15%)* |
|  |  |  | Alcohol | 10 *(0.94%)* |
|  | Cocaine | 242 *(22.78%)* | Amphetamine | 76 *(7.15%)* |
|  |  |  | Alcohol | 42 *(3.95%)* |
|  |  |  | 6-MAM | 12 *(1.13%)* |
|  | 6-MAM | 20 *(1.88%)* | Cocaine | 12 *(1.13%)* |
|  |  |  | Alcohol | 1 *(0.09%)* |
| **Amphetamines** | None | 167 | - | |
|  | Cannabis | 261 *(51.58%)* | Cocaine | 76 *(15.02%)* |
|  |  |  | Alcohol | 10 *(1.97%)* |
|  | Cocaine | 38 *(7.56%)* | Cannabis | 76 *(15.02%)* |
|  |  |  | Alcohol | 3 *(0.59%)* |
|  |  |  | 6-MAM | 1 *(0.20%)* |
|  | Alcohol | 6 *(1.18%)* | Cannabis | 10 *(1.97%)* |
|  |  |  | Cocaine | 3 *(0.59%)* |
|  | 6-MAM | 3 *(0.59%)* | Cocaine | 1 *(0.20%)* |
| **Cocaine** | None | 178 | - | |
|  | Cannabis | 242 *(53.78%)* | Amphetamine | 76 *(16.89%)* |
|  |  |  | Alcohol | 42 *(9.33%)* |
|  |  |  | 6-MAM | 12 *(2.67%)* |
|  | Amphetamine | 38 *(8.44%)* | Cannabis | 76 *(16.89%)* |
|  |  |  | Alcohol | 3 *(0.67%)* |
|  |  |  | 6-MAM | 1 *(0.22%)* |
|  | Alcohol | 29 *(6.44%)* | Cannabis | 42 *(9.33%)* |
|  |  |  | Amphetamine | 3 *(0.67%)* |
|  | 6-MAM | 7 *(1.56%)* | Cannabis | 12 *(2.67%)* |
|  |  |  | Amphetamine | 1 *(0.22%)* |
| **Heroin** | None | 34 | - |  |
|  | Cannabis | 20 *(43.48%)* | Cocaine  Alcohol | 12 *(26.09%)*  1 *(2.17%)* |
|  | Cocaine | 7 *(15.22%)* | Cannabis | 12 *(26.09%)* |
|  |  |  | Amphetamine | 1 *(2.17%)* |
|  | Amphetamine | 3 *(6.52%)* | Cocaine | 1 *(2.17%)* |
|  | Alcohol | 3 *(6.52%)* | Cannabis | 1 *(2.17%)* |
